# How do opt-in versus opt-out settings nudge patients toward electronic health record adoption? An exploratory study of facilitators and barriers in Austria and France

**DOI:** 10.1186/s12913-024-10929-w

**Published:** 2024-04-08

**Authors:** Anna Griesser, Manel Mzoughi, Sonja Bidmon, Emna Cherif

**Affiliations:** 1 Department of Marketing and International Management, University of Klagenfurt, Klagenfurt Am Woerthersee, Austria; 2 ICD Business School – LARA, Management Department, Lara, France; 3 Department of Marketing and International Management, University of Klagenfurt, Universitaetsstraße 65-67, Klagenfurt am Wörthersee, 9020 Austria; 4 University Clermont Auvergne, IAE Clermont Auvergne School of Management - CleRMa, Research Chair “Health and Territories”, Clermont-Ferrand, France

**Keywords:** Electronic health record, Opt-in and opt-out nudge, Barriers, Facilitators, Qualitative study, Austria, France

## Abstract

**Background:**

Electronic health records (EHR) are becoming an integral part of the health system in many developed countries, though implementations and settings vary across countries. Some countries have adopted an opt-out policy, in which patients are enrolled in the EHR system following a default nudge, while others have applied an opt-in policy, where patients have to take action to opt into the system. While opt-in systems may exhibit lower levels of active user requests for access, this contrasts with opt-out systems where a notable percentage of users may passively retain access. Thus, our research endeavor aims to explore facilitators and barriers that contribute to explaining EHR usage (i.e., actively accessing the EHR system) in two countries with either an opt-in or opt-out setting, exemplified by France and Austria.

**Methods:**

A qualitative exploratory approach using a semi-structured interview guideline was undertaken in both countries: 1) In Austria, with four homogenously composed group discussions, and 2) in France, with 19 single patient interviews. The data were collected from October 2020 to January 2021.

**Results:**

Influencing factors were categorized into twelve subcategories. Patients have similar experiences in both countries with regard to all facilitating categories, for instance, the role of health providers, awareness of EHR and social norms. However, we highlighted important differences between the two systems regarding hurdles impeding EHR usage, namely, a lack of communication as well as transparency or information security about EHR.

**Conclusion:**

Implementing additional safeguards to enhance privacy protection and supporting patients to improve their digital ability may help to diminish the perception of EHR-induced barriers and improve patients’ health and commitment in the long term.

**Practical implications:**

Understanding the differences and similarities will help to develop practical implications to tackle the problem of low EHR usage rates in the long run. This problem is prevalent in countries with both types of EHR default settings.

**Supplementary Information:**

The online version contains supplementary material available at 10.1186/s12913-024-10929-w.

## Introduction

Health Information Technologies (HIT) have revolutionized healthcare delivery and transformed the way patients and healthcare organizations manage personal health data [1]. By leveraging a variety of technologies including applications, Internet of Things (IoT), Personal Health Records (PHR) and Electronic Health Records (EHR), the implementation of HIT has enabled patients and healthcare professionals to store, share and analyze health data leading to improved outcomes, reduced costs, and enhanced patient engagement [2, 3].

One of the major HIT transforming the healthcare landscape is EHR, which serves as a centralized national repository for all patient data, including demographics, progress notes, past medical history, medications, vital signs, and immunizations [4]. The multitude of advantages arising from such technology from both patient and organizational perspectives derives from their ability to continuously collect, analyze, interpret, and disseminate health data. Specifically, the national Electronic Health Record (EHR)^1^ serves as a pivotal technology facilitating the exchange of health data among the three key entities: health policymakers, healthcare providers, and patients. By providing patients with access to their health information and diagnoses, it actively engages them in collaborative interactions with healthcare providers [5]. Despite the tool’s potential to improve patient care, increase resource efficiency, and reduce treatment errors, EHR adoption rates in many European countries have fallen short of government expectations [6, 7]. In Austria, for instance, 3.1% of the total population opted out of the EHR system in 2022, with deregistration numbers remaining constant since its introduction in 2015 [8]. Furthermore, in France, only 10 million EHR had been created three years after the generalization of the system, which is equivalent to 10% of eligible patients. Similarly, to Austria and France, in January 2023, Germany announced a number of users not exceeding 1% of insured patients [9].

Hence, previous studies have explored the factors that influence EHR usage from the patients’ perspective (i.e., [4, 10–15]). These studies have identified several facilitators, such as information incentives and training to improve patients’ digital literacy, perceived usefulness and user-friendliness, as well as high standards of security and privacy [16–18]. However, several barriers have also been identified that can impede patients’ adherence to using EHR, including privacy concerns, with the misuse of personal health data or lack of digital literacy being the main hurdles to broad acceptance of EHR [14, 19–21]. A recent study by Abbasi et al. [22] showed that, personal and technical readiness are among the most critical criteria affecting EHR usage.

Existing research has, nonetheless, mainly focused on explaining facilitators and barriers of EHR use and adoption through the general lens of the patient’s perceptions, thus neglecting one relevant factor that might significantly affect adoption by patients: whether the patient is a resident of a country with an EHR opt-out policy or an EHR opt-in policy. The opt-out policy entails automatic enrollment of all patients in the system, wherein personal health information is transferred to the EHR system unless patients choose to opt out. For instance, the Austrian EHR system offers patients the chance to opt-out if a person does not want to have an EHR. Consequently, in Austria, being considered a user is synonymous with not opting out, and actively accessing health records is not a prerequisite for being classified as a user [14]. However, refraining from opting out implies that physicians can utilize the EHR system for a specific patient, even if the respective patient is not actively involved in managing their EHR. If they do not opt out, his or her EHR can be accessed, at the very least, by Austrian healthcare professionals. Patient’s EHR usage in Austria, thus can vary according to the extent of usage reaching from passive usage to active and frequent usage. In contrast, in countries with an EHR opt-in policy like France, patients must expressly request the inclusion of their records. French patients need to actively opt-in to become users of the EHR system. In this context, being a user requires the explicit decision to participate and access health records [9]. Healthcare professionals, including physicians and pharmacies, utilize the EHR system in line with their professional roles, accessing and contributing to patient records as part of their clinical responsibilities [6].

Opt-in versus opt-out settings have gained increased attention in relation to many health-related issues (e.g., organ donations, obesity and overweight, chronic disease management, and vaccinations) in recent literature [23–25] as efficient interventions to (not) nudge and reinforce actions with a positive impact and deter actions with negative implications. Gong et al. (2020) show that the chosen mechanism depends on a personal perceived need and utility that impacts the decision [26]. As the use of an EHR system observably has positive contributions, measuring the stage of adoption throughout the country has become an important management tool for the health policy makers of the nations [27]. In line with these practices and in consideration of the high stakes for governments, understanding the underlying mechanisms of EHR adoption and use according to the type of policy (opt-in versus opt-out setting) is of high relevance for policy makers when deciding about implementing nudge interventions regarding EHR.

To date, research has not fully considered the impact of default settings in an opt-in versus an opt-out policy related to EHR systems on patient adoption. Liu et al. [28] showed that there has been no differentiation between opt-in and opt-out settings, despite a growing debate in the literature on the acceptability of the default nudge. Another study, however, claims that opt-out consent policies were associated with higher patient participation rates in EHR systems [29]. Additionally, Steinhauser and Raptis [30] found that the opt-out model resulted in a significantly higher participation rate, highlighting the impact of consent policies on patient engagement. To the best of our knowledge, our study is the first to fill this gap by exploring the mechanism underlying EHR usage in two EU countries with different system settings: France (opt-in) and Austria (opt-out) [31–33]. Understanding these exemplified differences, however, is also critical for other healthcare systems when selecting an effective strategy to boost EHR usage and improve patient care [32–34]. Nevertheless, it is imperative to clarify that the EHR under scrutiny in this study relates specifically to the patient-oriented solution rather than a standardized template employed by health providers. This distinction is pivotal in outlining our focus on the individual's interface with the EHR, clarifying the user-centric perspective that underscores our investigation.

### Theoretical foundation

#### The relationship between nudging and default settings from a psychological point of view

Nudges can be defined as “interventions that preserve freedom of choice but that nonetheless influence people’s decisions” ([35], p. 285). Whereas the theoretical principles of nudging have been thoroughly investigated in psychology during the past three decades [35] interest on the part of policy makers [36] and especially in healthcare settings [26, 36, 37] has only recently awakened.

One of the core tasks of legislatures is the decision to select nudges, amongst others in the form of default rules [28, 35, 38–40]. In the view of policy makers, designing government-imposed choice structures could influence peoples’ decisions in a helpful way. Defaults in the context of decision making as a specific form of nudges have been used to an increasing extent recently to improve approval for social policies. Especially in the context of vaccination policies (i.e., [41–43]), two types of policies exist. “An opt-in policy (vaccination is rejected by default; explicitly opting in is required if a person wants to be vaccinated) and an opt-out policy (vaccination is accepted by default; explicitly opting out is required if a person does not want to be vaccinated)” ([28], p. 2). Another area of applying nudging in the healthcare domain is, e.g., opt-out testing for blood-borne viruses (BBCs) as a method of case detection [44] based on positive results gained in the area of opt-out organ donation [45] and opt-out antenatal Human immunodeficiency virus (HIV) testing [46].To sum up, default settings only seem to be accepted for distinct purposes and without causing respondents to feel they are being manipulated. This draws attention to one central question, which is the definition of nudges as “subtle rearrangements of the choice architecture” ([35], p. 5), and points to the psychological roots of the functioning of nudges. In the realm of EHR systems, however, it can be assumed that the default setting of a government’s decision in favor of an opt-in or, alternatively, an opt-out system might have an impact on perceived facilitators and barriers to EHR usage of the respective population. However, different facilitators and barriers might prompt patients to actively access the EHR system in a country with either an opt-in or and opt out system.

#### The influencing chain of technology acceptance originating in the TAM

As EHR are a special form of innovative information technology, the general theoretical approach, i.e., the technology acceptance models, can be applied. The most widely known approach is the original Technology Acceptance Model (TAM), which was proposed by Davis in 1989 and suggested that two main factors influence a user’s decision about how and when they would use the new technology: the perceived ease of use (PEOU) and perceived usefulness (PU) [47, 48]. Besides external variables such as social influence, these two central determinants have an impact on the attitude toward a new technology and further the intention to use it. The basic model has been further developed by different authors leading to the TAM 2 [49, 50], the UTAUT [51], the TAM 3 in the area of e-commerce [52] and the most recent stage of theory development, the UTAUT2 [53]. The UTAUT [51], which proposed four central antecedents: performance expectancy, effort expectancy, social influence and facilitating conditions also accounted for moderating variables like gender, age, voluntariness and experience. The UTAUT2 [53] has become even more widespread and has added three new constructs to the influencing chain, i.e., hedonic motivation, price value and habit. For this study, it can be assumed that hedonic motivation can be neglected, because, first of all, EHR usage usually does not cause a financial burden for patients besides opportunity costs of life time which could have been spent doing other things rather than taking a look at the data saved in one’s own EHR. Secondly, considering that hedonic and utilitarian values are conceptualized as two types of knowledge drawn out of prior experience, the health category is related more closely to utilitarian value than to hedonic value [54]. Thus, we assume that performance expectancy, effort expectancy and social influence as well as facilitating conditions and habit might constitute possible antecedents of EHR usage.

#### Privacy calculus theory

Existing literature reveals that privacy concerns are among the most significant impediments to widespread adoption of EHR. Pang et al. [55] found out that, for patients, risk, control over access and trust are among the most important factors in terms of privacy concerns. Many patients are hesitant to take advantage of the EHR due to privacy concerns although they are eager to disclose personal health data to benefit from improved healthcare delivery [15]. When positive feelings and perceptions outweigh perceived risks and negative beliefs, privacy concerns can likely be alleviated [56]. For instance, previous research has shown that although risk perception may lead patients to engage in protective behaviors when benefits are high enough to override perceived risks [57–60], privacy concerns decrease, and intentions to use [58] and share personal health data increase [54, 61]. As evidenced by previous studies in the e-commerce context, consumers' personal positive and negative perceptions, such as privacy concerns, are related to inhibiting or driving behaviors. Inhibitors act as barriers to online transactions. By contrast, drivers encourage consumers to purchase online. The cumulative evaluation of the sets of inhibitors and drivers – privacy calculus – and the extent to which each set might outweigh the other influence the user’s final decision [55]. Thus, if the patient’s cumulative evaluation of the drivers to use EHR efficiently and disclose their personal health data is greater than their reluctance, they will more likely engage in the use and adoption behavior [62]. To sum up, based on the three theoretical frameworks explained, the following general research question was explored in our study:

- Which underlying mechanisms in the form of facilitators and barriers contribute to explaining EHR usage in countries with an opt-in vs. an opt-out setting?

## Methods

This research aims to examine specific facilitators and barriers contributing to explain EHR usage in opt-in and opt-out systems. EHR usage in our study refers to actively accessing the EHR system of the respective country and consequently a “user” in our study has been defined as a person who has accessed the EHR system at least once in the past.

### Country selection

In order to address these objectives, the research study examines two EHR systems: one used in Austria, known as ELGA, and the other used in France, known as DMP. These two European countries were selected for a cross-country investigation due to their similarities in terms of healthcare regimes.

**Fig. 1:**
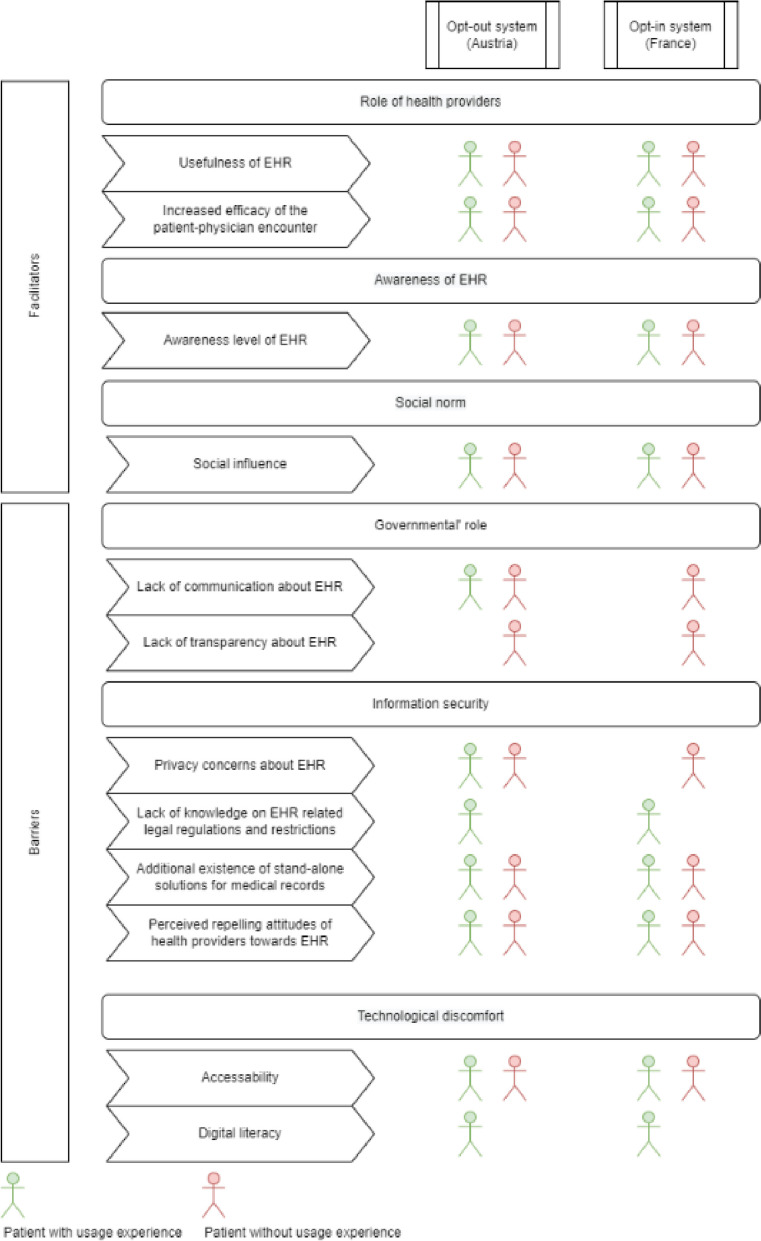
Code system of derived categories of facilitators and barriers in Austria and France

In Austria, the Electronic Health Record (EHR) system, established in 2009, operates under the legislative framework of the ELGA Act. Launched in 2015, the system interconnects various healthcare providers with the aim of enhancing information flow. Health data, generated by providers like hospitals and physicians, supplements medical treatments, and access to the EHR requires a multi-stage registration process via a personal mobile phone signature for identity verification. Patient control is emphasized, with the ability to block or remove attending health providers [14]. In France, the EHR is an optional and voluntary process for patients, regulated by the Act of Public Health Code. Patients create and manage their EHR, accessible online for medical monitoring. Access rights are stringent, with only attending physicians and Emergency Medical Services having full access. Patient consent is prioritized, allowing them to block or remove any health provider [9]. Both countries emphasize patient control and privacy, with distinct approaches to EHR governance and access rights [9, 14]. Figures 1 and 3 in Appendix (A) provide visual overviews of EHR governance in Austria and France, respectively.).

### Study design and data collection

To allow an in-depth understanding of patients’ facilitators and barriers when implementing EHR systems, a qualitative exploratory approach was taken with the help of an adapted interview guideline gathering the same themes for both samples. In pursuing these objectives, the research focuses on EHR systems in Austria and France. Although at first glance the two European countries appear similar in terms of health care regime, they are currently very different with respect to cultural values and dimensions as well as EHR system default settings.

On the one hand, Austria places great value on an egalitarian social structure. According to Hofstede Insights [63], Austria scores very low on the “Power Distance” dimension, meaning that emphasis is placed on participative communication and equality among members of society. The social welfare system of Austria reflects this value of equality. Furthermore, the Austrian propensity towards warmth is manifested through their ways of socializing with one another. For example, Austrians often enjoy engaging and learning through conversation and socializing in public places is also common. Furthermore, citizens are automatically enrolled in the EHR system, resulting in a higher overall awareness and familiarity with the system. Given this heightened awareness among the Austrian population, focus groups were deemed a suitable method, allowing participants to engage in group discussions and share their experiences more openly, leveraging their collective knowledge.

On the other hand, France scores fairly high on the “Power Distance”, which indicates the manner of communication is determined by social status, level of education, and age. Tone and choice of words will vary among these factors [63]. The French population, enrolled in the DMP system, faces a different dynamic due to the opt-in nature of the program. This results in potentially lower awareness among citizens. The decision to conduct individual interviews in this context aimed to provide a more focused and personalized exploration of participants' experiences, compensating for potential disparities in group engagement. Thus, the French opt-in enrollment prompted individual interviews to delve into personal experiences while the Austrians’ automatic enrollment might lead to higher EHR awareness, making focus groups conducive to open discussions. The primary rationale for adopting distinct methodologies was both grounded in the concept of “power distance” and rooted in each national context’s unique characteristics.

In order to make the results comparable, the same semi-structured interview guideline was developed and coordinated in several feedback loops within our research teams and can be found in the Appendix (B). In Austria, four online focus group discussions were conducted in September and October 2021, leading to an average length of 60 min per focus group. In France, the data collection started in November 2021 and lasted three months, covering nineteen individual online semi-structured interviews, also with an average length of 60 min. Concerning the study’s sample size, research subjects, and quality of responses, semantic saturation was achieved due to the plateau of the emergence of new themes and codes [64].

### Analysis procedure

In a first step, the two research teams in Austria and France developed a first codebook independently for each country. In the next step, codings were discussed, and consensus was reached to develop a common codebook. During this coding process, coders worked independently to reveal any similarities or discrepancies with regard to EHR adoption, first using Excel spreadsheets, which were transferred into MAXQDA after data cleansing procedures. Data were sorted across participants and country of origin and then categorized into subcategories according to the four codes: barriers and facilitators, each assigned to either the opt-in system or the opt-out system. In order to obtain results that are robust and valid, we adopted a meticulous approach to data analysis. Specifically, we categorized the codes associated with barriers and facilitators into distinct types and cross-referenced the data according to the type of regime. Finally, to ensure the validity of the analysis, an independent reviewer checked the data and confirmed the results’ convergence. To organize the source data more clearly, codes were classified into main codes and subcodes within MAXQDA. Specifically, EHR facilitators and barriers served as two overall main codes, which can comprise numerous subcodes, each containing their own subcodes. The results presented are the most frequent categories that emerged from the data.

### Participants

As this study aims to examine similarities and differences between an opt-in and an opt-out regime of EHR, our target population consists of citizens who are potential or actual patients. In the Austrian sample, the designation of 'non-user' pertains to patients who, although not having actively opted-out, have not accessed their EHR system in the past. Conversely, the term ‘user’ is assigned to patients actively engaging with the EHR system. To elaborate on the French system, ‘users’ are defined as patients who have deliberately opted in and are actively accessing the system. At the same time ‘non-users’ comprise those who are not presently accessing the system. Participants are not limited to residents of hospitals and clinics, ensuring a balanced sample. Furthermore, the recruitment strategy aimed to capture a spectrum of demographics, including age, gender and education levels, to enhance the generalizability of findings to the broader citizenry. Participants were drawn from various backgrounds, encompassing both urban and rural settings, to mitigate potential biases. The four groups comprised a total of *N* = 30 participants from Austria and were homogenously composed with regard to user experience and age (see Table 1). The same sampling conditions were applied when recruiting the *N* = 19 French participants (see Table 2).

**Table 1:** Austrian sample description separated according to subgroups | discussion groups of EHR Users [groups 1 and 2] vs. non-users [groups 3 and 4]

**EHR Users (Participants with previous EHR usage experience)**									
**Group 1: EHR Users aged up to 45 years**					**Group 2: EHR Users aged 46 years and above**				
ID	Gender	Age	Highest level of education	EHR usage experience in years ^a^)	ID	Gender	Age	Highest level of education	EHR usage experience in years^a^
U1	female	29	University	2 (2019)	U8	male	52	High School	6 (2015)
U2	male	30	University	< 1 (2021)	U9	male	47	High School	1 (2020)
U3	female	33	University	3 (2018)	U10	male	70	High School	7 (2014)
U4	female	25	University	3 (2018)	U11	male	53	University	2 (2019)
U5	female	26	University	< 1 (2021)	U12	male	61	University	6 (2015)
U6	female	29	High School	6 (2015)	U13	male	55	University	< 1 (2021)
U7	male	26	High School	< 1 (2021)	U14	female	46	University	2 (2019)
					U15	female	57	High School	7 (2014)
					U16	male	47	University	6 (2015)
**EHR Non-users (Participants with no previous EHR usage experience)**									
**Group 3: EHR Non-users aged up to 45 years**					**Group 4: EHR Non-users aged 46 years and above**				
ID	Gender	Age	Highest level of education	EHR usage experience in years 1)	ID	Gender	Age	Highest level of education	EHR usage experience in years^a^
N1	male	35	University	none	N8	male	55	University	none
N2	female	29	University	none	N9	male	54	University	none
N3	female	23	University	none	N10	male	68	University	none
N4	female	27	University	none	N11	female	50	High School	none
N5	male	25	University	none	N12	female	46	University	none
N6	male	21	High School	none	N13	male	52	High School	none
N7	female	29	University	none	N14	male	61	High School	none

^a^The year of the first access to the EHR system in Austria was included in brackets

**Table 2:** French sample description separated according to subgroups | interviews of EHR users versus non-user

**EHR Users (Participants with previous EHR usage experience)**				
ID	Gender	Age	Highest level of education	EHR usage experience in years^a^
U1	male	47	High School	6 (2015)
U2	male	24	High School	1 (2020)
U3	male	70	High School	7 (2014)
U4	male	53	University	2 (2019)
U5	male	61	University	6 (2015)
U6	male	28	University	1 (2021)
U7	female	46	University	2 (2019)
U8	female	57	High School	7 (2014)
U9	female	34	University	6 (2015)
**EHR Non-users (Participants with no previous EHR usage experience)**				
ID	Gender	Age	Highest level of education	EHR usage experience in years 1)
N1	male	55	University	none
N2	male	54	University	none
N3	male	68	University	none
N4	female	50	High School	none
N5	female	46	University	none
N6	male	52	High School	none
N7	male	61	High School	none
N8	Female	28	University	None
N9	Female	39	University	None
N10	Female	33	High School	None

^a^The year of the first access to the EHR system in France was included in brackets

The ethical boards installed in each of the two universities of the research teams in Austria and in France gave their consent to the data collection procedures ahead of data collection.

## Results

Utilizing the recommended approach of qualitative content analysis according to Mayring [65, 66], this study analyzed facilitators and barriers for two distinct patient groups – those with and without prior experience of using EHR systems – across two countries, one with an opt-in policy (France) and the other with an opt-out policy (Austria). The analysis identified facilitators and barriers organized into subcategories, such as the role of health providers and social norms, as well as the governmental role, information security, and technological discomfort. The results of this analysis are presented in the following themes, which arose from the interview guide (Fig. 1).

### EHR facilitators

#### Role of health providers

##### Usefulness of EHR

The usefulness of EHR is a high-priority topic of discussion in the study, and the analyses indicate that there is a positive trend towards increased usage among patients with prior experience, which has been reinforced by the COVID-19 pandemic. One participant from Austria highlighted the benefits of EHR beyond simply storing personal health data, stating that they can also be used for additional features such as the COVID-19 electronic immunization record. *“The benefit is not only in the triage of all personal health data, but also in the additional features one can use, such as the COVID-19 electronic immunization record.”* (Austria, U12, Male, 61]). In addition, some patients with no prior usage experience express *“…that the information is trustworthy and verified by a physician.”* (Austria, N1, Male, 35).

The clear majority of French participants agreed that being integrated in a medical digitalization system cannot be beneficial without multiplying the efforts and contacts. Their perspective suggests that the potential benefits of EHR outweigh the additional burden that may come with using them. They recognized the value of EHR for improving healthcare delivery and patient outcomes. Hence, we can cite the relevant quotes *“I do not doubt that we need to accept the need to share and use EHR; if the system is not at risk of being dismantled, as was the case a few years ago.”* (France, N1, Male, 55).

##### Increased efficacy of the patient-physician encounter

The health provider’s ability as gatekeeper to influence EHR usage was classified as that of a facilitator who motivated patients, as the following quote illustrates: *“I see the physician as the best option to convince patients to use EHR systems, for instance, concerning positive communication incentives after an examination: Today, you had your blood test, and tomorrow you can already view your results in the EHR system.”* (Austria, U10, Male, 70). In another context related to the opt-out regime, patients described behavioral loyalty to their health provider due to a feeling of bonding. “*The patients trust physicians and if it works at the grassroots level, it gets transported upwards. This is an opportunity that should be used*!" (Austria, U6, Female, 29).

Obviously, completed EHR are able to help health providers to improve communication with patients, especially when tracking patient progress over time*.* Participants report that a strong emotional connection can be developed due to trust and confidence in their physicians*. “My attending physician promptly diagnosed my cancer through the centralization of my previous medical examinations. I consider myself fortunate to have had such a knowledgeable medical professional overseeing my care”*. (France, U9, Female, 34).

#### Awareness of EHR

##### Awareness level of EHR

The awareness of EHR usage from the patients’ perspective does not only positively influence the individual treatment process, but even unnecessary additional treatments and time efforts for the healthcare system are seen by patients (“*I am thinking of multiple treatments [laboratory, X-ray, *etc*.], long waiting times for results, *etc*. The data simply transfers from one place to the other without any major problems*.” (Austria, U12, Male, 61)).

Both users and non-users of EHR in France expressed that increasing their awareness of the benefits they can gain from utilizing EHR has a positive impact on their intention to share personal health data. Several participants mentioned that they received information about EHR, which contributed to their understanding of the system and its advantages: *“The first time it was in 2017 that I received an e-mail from the social security organization expounding the new system and how to create one’s own EHR. After that and whenever I was sick, my doctor used to give me an informative flyer about the EHR functions.”* (France, U3, Male,70).

#### Social norm of EHR

##### Social influence

Without the support of, for instance, family members, friends or health professionals, patients, especially older patients, might be limited during the EHR usage process. In addition, during the registration process there is a steady need for assistance. (*"An important factor is certainly the social component of support during the EHR usage.”* (Austria, U13, Male, 55)).

For non-users in France, there is a notable and increasing social demand for engagement in a medical information sharing system. Participants are frequently faced with encounters with health providers or their relatives, which further emphasizes the significance of their involvement. “*It would help my attending doctor, the other care-providers and myself* *to be up to date with my healthcare. This task is a personal responsibility if we want to make things change.”* (France, U7, Female, 46).

### EHR barriers

#### Governmental ‘role’

##### Lack of communication about EHR

The lack of information emerges as a significant barrier to the utilization of EHR. This barrier encompasses various aspects, including insufficient knowledge and understanding of EHR systems among potential users. Therefore, one of the primary challenges faced by patients is a lack of awareness regarding the benefits and functionalities of EHR. At the very beginning of the rollout plan patients remembered negative propaganda (i.e., flyers, posters, etc.) with an invitation to opt out of the entire EHR system (*“Get out of the EHR system”* (Austria, N1, Male, 35)). Nevertheless, the current information available on EHR was still perceived as insufficient. (*“A big area is undoubtedly the information gap that exists. Patients do not know exactly what an EHR system is, what benefits it brings or what to do with it.”* (Austria, N3, Female, 23)).

However, many non-users stipulate that they are not adequately informed about how EHR can enhance the quality and coordination of healthcare, improve patient outcomes, and streamline medical processes. Without a clear understanding of these advantages, many patients still hesitate to adopt EHR and perceive them as unfamiliar or unnecessary. (*“It was clear that I wouldn’t be enrolled, however even the social security organisation paid no attention at all and didn’t even follow up with me to join the EHR system.”* (France, N6, Male, 52)).

##### Lack of transparency about EHR

The lack of transparency surrounding EHR significantly hampers their adoption and utilization. Consequently, being well informed about the advantages of health digitalization is the best way to enhance use of EHR in both the opt-in and the opt-out regime. In Austria, patients are not aware of the opt-in setting and the process of ‘opting-out’ (*“…It’s interesting that you have to go through the registration process before you are allowed to opt out”* (Austria, N9, Male, 54)).

Similarly, in France, the lack of transparency regarding EHR contributes to a lack of awareness among patients. Some participants pointed out the lack of clear and accessible information leading to a lack of understanding, confusion and mistrust. This lack of information becomes evident in statements of patients, for instance, *(“Why should I risk my data privacy? Health organization language is very vague.”* (France, N9, Female, 39)).

#### Information security

##### Privacy concerns about EHR

Bearing the sensitivity of the exchanged data in mind, patients found that data with regard to privacy security throughout the entire usage process had to be comprehensively guaranteed. Especially, possible restrictions/regulations by the patient of their sensitive data were addressed repeatedly. Nevertheless, a kind of uncomfortable feeling emerged in the form of (*“You still feel like a human being made of glass.”* (Austria, U12, Male, 61)).

Some French participants questioned the protection of data during the collection level, the transmission levels, and the storage level: (*“I am not able to face the scourge of data privacy. I am unable to prevent my personal data from being disclosed; we hear about it every day i.e. Facebook scandal; and no one can stop this process.”* (France, N8, Female, 28)).

Patients express that privacy security guarantees of the EHR system seemed to be sufficiently implemented through the complex and secure, but challenging login procedure. However, if a data leak appears, the impact is immense and, in the worst-case scenario, (*“ If people can access my data and use it against me, this could lead to social exclusion due to, for example, mental illness, delayed procedures for applying for early retirement, or job loss.”* (Austria, U12, Male, 61)).

##### Lack of knowledge on EHR related to legal regulations and restrictions

None of the patients has in-depth background knowledge of whether, and if so, which specific law (i.e., European basic data protection regulation) is behind the EHR system in Austria. Only one patient with usage experience confessed that the situation was unclear for the parties involved since (*“ each health institution defines for itself what goes into the EHR system (i.e., doctor’s letter, laboratory, X-rays, *etc*.], although this is actually required by law in Austria.”* (Austria, U8, Male, 52)).

Similarly, French interviewees display a lack of awareness that EHR are a standardized and secured digital health record implemented by the Ministry of Health. Participants completely ignore the regulatory aspects of EHR, including important ones, despite the French government’s efforts, for example insisting on the consent that gives patients the possibility to block or remove any health provider from the list at any time. Hence, none of the participants mentioned this regulation. (*“I can’t share all of my medical history for fear that my employer will fire me after realizing that I have a chronic illness”.* (France, U4, Male, 53)).

##### Additional existence of stand-alone solutions for medical records

Especially in Austria, the existence of additional stand-alone solutions for medical records might add confusion as (*"in some hospitals in, for example, other federal states, the examination result is provided electronically and in other facilities it continues to be provided in analogue form."* (Austria, U10, Male, 70)). However, non-users address that with the co-existence of additional stand-alone solutions for medical records, the perceived usefulness of an EHR system on a national basis is raised into question. ("*As long as there are different systems from, for example, private institutions, there is also no added value.*" (Austria, N5, Male, 25)). French users and non-users of EHR figured out that standardization of EHR systems between public hospitals, private institutions and physicians is a priority to achieve more efficient infrastructure. *(“When asking for a medical check-up I often encounter a frustrating experience where they have their own separate online systems or I switch to the “doctolib” application.”* (France, N1, Male, 55)).

##### Perceived repelling attitudes of health providers towards EHR

From the patients’ perspective however, a repelling attitude of health providers towards EHR was reasonable. It was attributed to an increased additional effort in terms of the financing of and training in the use of the EHR system, a burden imposed on the health providers (“*…in the form of a new system, training and education for health providers, maintenance and support, *etc*.”* (Austria, N1, male, 35)).

Thus, physicians are the primary users of EHR systems, and their engagement and buy-in are critical for the successful implementation and adoption of the system. Many participants reported that they will cross the line only if their attending health provider guarantees the secure handling of their data: (*“I have recently completed my EHR, and still panic about the idea that my data can be hacked. Unfortunately, none of my health providers explained to me the importance of sharing my medical data or reassured me that in case of emergency only health providers would have access.”* (France, U1, Male, 47)).

#### Technological discomfort

##### Access to and completion of the EHR

Patients, regardless of their usage experience, consistently emphasized the importance of having an EHR system that is user-friendly, easily understandable and tailored to their specific needs. This is seen as a crucial factor in increasing the likelihood of widespread adoption and high levels of utilization. One statement highlights the frustration caused by a poorly designed and unclear platform: *“The platform itself is totally unclear and badly designed, so it is not understandable, which wastes a lot of my time with every log in".* (France, U1, Male, 47)).

In Austria, the technical necessity (i.e., personal digital identification via smartphone) for secure access and a transparent usage process was perceived as an indispensable albeit challenging factor. A system that is easy to understand and tailored to patient needs is seen to increase the chances of a high level of system use. As mentioned by patients (*“The login process, with its path and various links and shortcuts, is much too lengthy and complicated.”* (Austria, U6, Female, 29)).

##### Digital literacy in relation to EHR usage

The theme ‘digital literacy’ refers to the specific competency necessary to be able to manage and use EHR effectively (*"Many, unfortunately, are not ready yet, the digital competency is lagging here."* (Austria, U2, Male, 30)). In general, digital literacy was identified by experienced and *“younger French users as a prerequisite to handle the EHR system”* (France, U2, Male, 24) to be able to navigate through the EHR system (*"People who use EHR systems can also more easily use similar platforms (i.e., mobile banking).”* (Austria, U9, Male, 47)). Dealing with technological discomfort experienced by participants placed an emphasis on the role of media and the key stakeholders of healthcare delivery in assisting patients in need, especially the elderly. Indeed, young interviewees express being creative in developing innovative and useful features of EHR systems, for instance, (“*integrating the vaccination status and generating automatic reminders for having a booster shot*”, (France, U2, Male, 24)).

## Discussion

### Contribution to theory

This research aims to contribute to the expanding literature on the facilitators and barriers to EHR adoption, specifically comparing the opt-out system in Austria to the opt-in system in France. We find that patients in both countries have similar experiences regarding the facilitating factors, such as the role of health providers, awareness of EHR, and social norms.

Our results are consistent with previous work in this field highlighting that perceived usefulness [48, 49], including up-to-date and complete health information, can be identified as a key factor in increasing EHR usage in the long run [20, 67]. Especially heavy consumers of the healthcare system due to multimorbidity and high intensity of healthcare services could be classified as EHR supporters in the study by Halmdienst and others [14, 68]. Their study also showed that patients with an optimistic attitude or enjoyment of life have a positive impact on using EHR. Furthermore, the provision of system training or experts/power-users to work with the EHR system were considered to facilitate EHR usage [22]. Our in-depth view shows that this relationship might be explained at least to a certain degree by increased efficacy of the patient-physician encounter due to EHR usage. This finding corroborates the results of Abbasi et al. [22], who identified usability as one of the most important reasons for using the Iranian EHR system SEPAS from the end-users’s perspective.

Furthermore, our study also reveals that physicians play a significant role as facilitators and gatekeepers by motivating patients and increasing their awareness of EHR [69–71], which is also in line with existing literature assuming enhanced IT competencies and confidence regarding EHR systems by physicians [72]. This might go hand in hand with an increased awareness of health prevention and promotion (i.e., health screenings) of patients, which was found to contribute to higher EHR acceptance in existing literature on EHR usage [14].

A high degree of social systems and connectedness, influence by family, friends and health providers, were found to be essential for EHR usage and generate spillovers to provide access to EHR regardless of location and time [3, 14, 18, 73]. Without the support of others, patients may face limitations when handling the complex, though classified as secure, login procedure and in their ability to navigate through the features. Empowering patients from a societal perspective, especially when belonging to the elderly population, is critical, as Torrens and Walker [73] point out. Patient empowerment can help to improve the acceptance and adoption of EHR or other patient portals and can assist in fulfilling other daily – digital – tasks [14].

However, participants’ views are divided in Austria and France regarding the barriers to EHR usage. The lack of communication and transparency about EHR on the part of the government is identified as a major barrier in both countries. In France, uninformed patients expressed concerns about actively disclosing their EHR data, highlighting the importance of patient awareness in decision-making. Additionally, information security is considered as a prerequisite in both countries, with patients’ motivation for better health outweighing their privacy concerns. Patients without usage experience in France express the greatest concerns regarding privacy, indicating a different information situation compared to patients with usage experience [19, 69]. On the other hand, lack of knowledge about government regulations and restrictions related to EHR is noted among patients who have already interacted with the system in both countries.

In line with previous research [19, 20] on privacy concerns and data protection, information security was identified as a necessary prerequisite in both countries, especially the setting in which it is implemented by the federal authorities – opt-in or opt-out. In line with existing literature [20], and based on the privacy calculus theory [38] we found that patients’ motivation [19, 39, 40] for better health clearly overrides their concerns and boosts their confidence that privacy is being respected by the government [15]. Thus, our study’s findings show again that French patients without usage experience express the greatest concerns regarding privacy due to a different information situation compared to patients with usage experience. Furthermore, a lack of knowledge of the government’s EHR-related legal regulations and restrictions [8, 9] is only noted among patients who had already dealt with the EHR system in both countries. Liu et al. [28] demonstrate that participants show relevant motivation to get involved in an EHR rollout plan. This complexity highlights the existence of barriers that hinder patients from actively using EHR and that has not been analyzed in-depth in other literature before. Austrian and French patients mention additional stand-alone solutions for medical records provided by private hospitals and physicians as frustrating regarding the inconsistency of data from multiple sources, which are not always compatible with one another.

In both countries, patients perceive a negative attitude from health providers towards EHR, emphasizing the importance of health providers’ motivation and commitment to EHR adoption, which aligns with previous studies [62, 64]. This statement clearly shows that the health providers’ motivation and commitment determine whether an EHR system is used or not [18, 19, 64]. Thus, physicians can be seen as noticeable gatekeepers [6]. Finally, technical discomfort, including lack of accessibility and digital literacy, are identified as a significant challenge in both countries, affecting patients’ ability to use the system effectively [3, 19, 20].

### Contribution to practice

Our results corroborate the findings of a study by Crameri et al. [74], who found that one of the most significant barriers to EHR usage is a lack of patient communication about the technology. Thus, nationwide and standardized communication measures initiated by the government can be used, which may ultimately help to increase and maintain patients’ EHR handling process [2]. Köse et al. [27] suggest including EHR communication strategies in form of interpersonal, interactive, and mass media (i.e., login procedure, opt-out scheme, etc.) as a target in national strategic plans, which have been identified as vital encouragements of EHR usage in their study. Also, Abbasi et al. [22] pointed out the implication of changing the philosophy from local to national comprehensive plans to integrate a working interoperable EHR system. From a macro perspective, boosting EHR usage by communication measures might contribute to a government’s higher degree of additional information supply that might not be evaluated in a trial or study. The data collected during and after a treatment process enable the evaluation of outcomes and resources across patients’ long-term interactions with the national healthcare systems (i.e., medications, health services, diagnoses) [65]. This subject could also be included on the meso-level of communication, demonstrating that health providers are aware and actively address the topic of EHR education and training [71]. The findings of our study corroborate the findings of Halmdienst and others [14], revealing that some patients have a low digital literacy level due to a lack of familiarity with health IT resulting in an insufficient understanding of functions and usefulness of EHR. It may be beneficial to encourage social contacts (i.e., family, health provider) to support patients along the digital path. Considering that findings have indicated that the concerns regarding information security may be a matter of individual choice, government could make patients more aware of their EHR related legal regulations and restrictions [3, 8, 9]. Mediated health communication to reduce barriers and/or foster facilitators by using a segmentation approach (users vs. non-users) could boost EHR usage [16].

In light of the foregoing, to improve EHR adherence regardless of the regime type, multiple interventions inspired by the nudge theory have to be employed [1]. Our research brings to light a shared concern prevalent in both settings, a noticeable lack of awareness among patients regarding their opt-in or opt-out status, stemming from deficiencies in information dissemination and transparency [19, 69]. Patients, irrespective of the governmental opt-in or opt-out setting, expressed a lack of awareness regarding the registration process and subsequent opt-out options in Austria; similarly, in France, a lack of clear and accessible information contributed to a shortage of understanding and heightened mistrust. The discussions encompass patients’ apprehensions about information security, confusion arising from unclear and inaccessible information, and the need for improved communication strategies [15, 16]. In this context, it is crucial for health policymakers to actively engage healthcare providers in the early stages of deliberation on the implementation and dissemination of EHR. Additionally, they must determine the appropriate communication strategies for informing patients about the accessibility and usage of health data. Contrary to viewing digitalization as a standalone solution for healthcare challenges, it should be seamlessly integrated into existing processes that involve the primary stakeholders in healthcare delivery. As evidenced by previous research on the healthcare provider relationship, healthcare professionals play a pivotal role in serving as ambassadors for EHR among patients [6, 69]. Healthcare providers emerge as critical figures in fostering EHR adoption and, notably, in establishing a trustworthy relationship with patients. In fact, this requires setting EHR functionalities and features to default options that promote adherence. For example, pre-selecting relevant options or templates for data entry can guide users towards providing comprehensive and accurate information, reducing the likelihood of errors or omissions [2].

However, the concept of nudging, popularized by behavioral economics, has been applied to various policy interventions beyond EHR adoption [35]. In the context of donor registration, nudging approaches have been implemented to increase organ donation rates. This opt-out approach has shown success in increasing donor registration [23]. Similarly, nudging techniques have been employed in chronic disease management to encourage healthier behaviors among patients. These interventions often involve personalized feedback, reminders, and incentives to promote adherence to treatment plans and lifestyle changes [25]. In the context of vaccinations, timely reminders, social norms, and simplifying the vaccination process aim to influence individuals’ decision-making [41, 42]. Comparatively, the adoption of EHR systems involves distinct challenges and considerations. Unlike some nudging interventions, EHR adoption is not solely reliant on individual behavior but involves complex interactions between patients, healthcare providers, and policy frameworks. Understanding the differences and similarities between nudging approaches in various domains and EHR adoption can provide valuable insights into the effectiveness of different behavioral interventions and their implications for public initiatives [75].

Moreover, to nudge users towards desired actions, national organizations have to implement timely reminders and prompts within the EHR system. These reminders can be designed to prompt clinicians to update patient records, complete specific tasks, or follow best practices [3]. By simplifying choices within the EHR interface, the patients’ decision-making process could be streamlined. This can be achieved by presenting information in a clear and concise manner, avoiding overwhelming options or complex navigation. Patients will then be more likely to adhere to the EHR workflow and make informed decisions efficiently [4]. Finally, it will be very efficient to incorporate social norms and peer influence within the EHR system to promote adherence. By highlighting the positive behaviors of colleagues or presenting aggregate data on adherence rates, medical authorities can create a sense of social pressure and motivate users to align with desired practices. Leveraging the power of social influence can encourage users to conform to the norms and standards established within the EHR environment [8, 76].

## Conclusion

This study provides an understanding of patients’ facilitators and barriers when using EHR systems, in the context of two different policies with an opt-in (France) and opt-out (Austria) setting. The results reveal very few differences in facilitators and barriers between the two countries. Significantly, the opt-in model implemented in France has led to lower rates of usage when compared to settings that prioritize enhanced privacy protection and incorporate additional safeguards [55]. In our view, the Australian My Health Record system (MHR), Australia’s national EHR system, might be seen as a best practice example combining the advantages of both the opt-in and the opt-out settings. The initial opt-in setting in Australia prompted only 2 million registrations. After switching to an opt-out setting, more than 23 million Australians, i.e., 90% of Australians, had an EHR in 2019 due to the strength of privacy protection and additional safeguards [77]. Hollo and Martin [77], however, put emphasis on the promotion of equity in health information security for EHR users. Thus, we highlight the importance of recognizing the potential benefits of implementing similar safeguards by, at the same time, considering equity in health information security in France to encourage higher adoption rates of EHR. Furthermore, Austria, lacking comparable safeguards, might benefit from exploring similar strategic options. In summary, each country should tailor its system's framework according to its national requirements. In the event of any deviations during the implementation phase, governments must take swift and effective action to address them [72]. An essential aspect of successful implementation lies in the adaptability of the roll-out plan to address unforeseen deviations. This responsiveness is vital to maintaining the integrity of the system, fostering public trust, and ultimately achieving the intended positive impact on healthcare outcomes and patient experiences.

### Limitations and recommendations for future research

The authors acknowledge limitations, such as the qualitative approach focused on comparing only two countries. Hence, this research can be viewed as an initial step in comprehending different EHR regimes, providing valuable insights into the facilitators and barriers. However, to establish more robust and generalizable findings, it is imperative to expand the scope of the study by incorporating a larger sample of European countries such as Germany or Sweden. The participants’ disproportionate distribution in terms of educational background may reduce the external validity of the results. Future research could address these limitations by employing a larger and more representative sample. Furthermore, the participant selection process does not consider individuals who actively opted out due to a strongly negative disposition towards EHR. Those people may bring further insights on the perspectives of those opposed to EHR adoption. Thus, future research is encouraged to address this specific subgroup of people who have actively opted out of the EHR system in a country with an opt-out setting. Considerably more work will need to be done to determine the specific motives and reasons of those who have already opted out in order to contribute to a nuanced comprehensive understanding of attitudes and barriers related to EHR adoption by focusing on the other side of the coin, which is the refusal to participate in EHR systems.

Our research has also given rise to several questions in need of further investigation. If the debate is to be moved forward, a better understanding, especially of the needs of the elderly, needs to be developed. Against the backdrop of the aging of society, it should be mentioned that older adults often face increased personal health concerns and suffer from multimorbidity. To an increasing extent, this makes it crucial to understand how EHR can address their specific needs and enhance their healthcare experience. What is more, elderly patients may encounter difficulties in using new technologies, including EHR systems, due to factors such as limited digital literacy, unfamiliarity with technology, and potential physical or cognitive limitations. Overcoming these challenges in the realm of EHR is an important issue for future.

### Data availability

The full data set and materials pertaining to this study can be obtained from the corresponding author on request.

### Supplementary Information

**Supplementary Material 1. **

**Supplementary Material 2. **

## Data Availability

The authors confirm that the data supporting the findings of this study are available upon request.

## Footnotes

1 The terms "Personal Health Records" (PHR) and "Electronic Health Records" (EHR) refer to distinct concepts in the realm of health information management. A Personal Health Record (PHR) typically involves health data managed by individuals, allowing them to store, track, and manage their health information. On the other hand, Electronic Health Records (EHR) encompass digitalized patient health records maintained by healthcare providers, facilitating comprehensive and centralized access to patient data within a healthcare institution [5].
