# Supplementary material for: How do opt-in versus opt-out settings nudge patients toward electronic health record adoption? An exploratory study of facilitators and barriers in Austria and France
Source: BMC Health Serv Res. 2024 Apr 8;24:439. doi: 10.1186/s12913-024-10929-w (PMC11003073; doi:10.1186/s12913-024-10929-w)
Supplement: Supplementary file 1 — Supplementary Material 1. [file 12913_2024_10929_MOESM1_ESM.docx]

**Overview of EHR in Austria**

The principals in Austria (federal, state, and local governments) established the EHR system in 2009. In order to legally secure their actions, a legislative basis was created in the form of a special legal section (ELGA Act), Art. 15a of the Federal Constitutional Act on the organisation and financing of the health care system, specifically the foundation on an electronic health record (ELGA). The actual project started in 2015 with the intention to interconnect different health provider of the entire healthcare system (patients, hospitals, physicians, care facilities, pharmacies, etc.). Health data such as a patient’s test results are generated by a variety of health providers. Its aim is to supplement medical treatments and consultations with improved information flows, particularly when several service providers are collaborating. In order to have access to a specific EHR via the portal, a multi-stage registration process with the help of one’s personal mobile phone signature (“Handy Signatur” in German) has to be completed. This electronic ID enables the EHR system to verify the individual patient’s identity and ensures data protection. Furthermore, the system ensures that solely attending providers (i.e., physicians, pharmacies, hospitals) can access EHR content during an ongoing course of treatment or health care. Moreover, to safeguard information security, physicians working for state institutions (i.e., government offices, insurance companies, company medical services) are prohibited to access EHR. Meanwhile, patients have the right to block or remove any attending health provider from access to their own EHR. Figure 1 gives an overview of EHR governance and exemplary access rights in Austria.


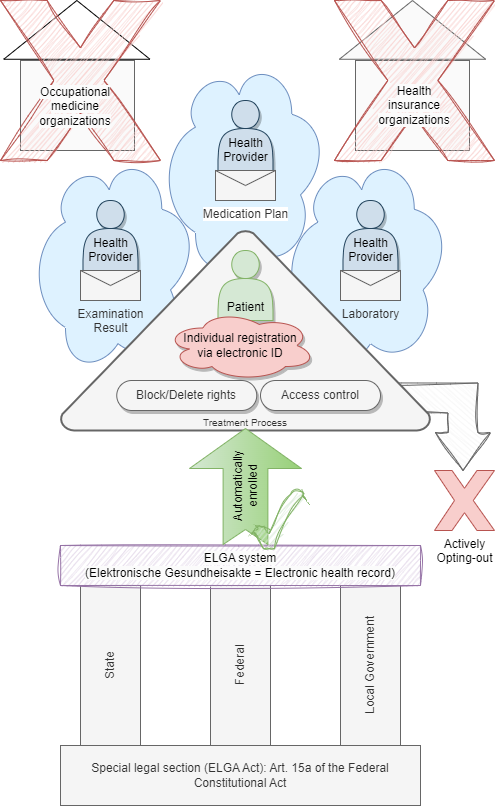


Figure S1: Systematic overview of EHR governance and exemplary access rights in Austria

**Overview EHR in France**

French authorities have defined a specific legal framework to encourage EHR dissemination and adoption by French users. Thus, the section L. 1111-11 of the Act of Public Health Code states that the creation, as well as the closure, of an EHR is an optional and voluntary process to be undertaken by the patient himself. EHR is a free, standardized and secured digital health record provided by the Ministry of Health that may bring together all patient personal health information. Patients can create, through the website or the mobile application, their own EHR. Moreover, patients can access their EHR online at any time to share and supplement it with health information for their medical monitoring such as medical history (chronic pathology, allergies, etc.), results of examinations and hospital reports, and contact details of their loved ones to be notified in case of emergency. The information shared allows both health providers to improve patient care and reduce costs (better informed about the medical history and the treatments followed, they act more quickly and avoid prescribing unnecessary or redundant examinations), and patients to have all their medical information on a single platform in order to be better taken care of by Emergency Medical Services in case of an immediate risk of survival. According to French law, only attending physicians and Emergency Medical Services are allowed to access the entire EHR content, other health providers must ask for the permission. Moreover, to avoid privacy issues, health insurance and occupational medicine organisations are prohibited to access EHR data. Finally, the French government insists on the consent that gives patients the opportunity to block or remove any health provider from the system. Figure 3 gives an overview of EHR governance and exemplary access rights in France.


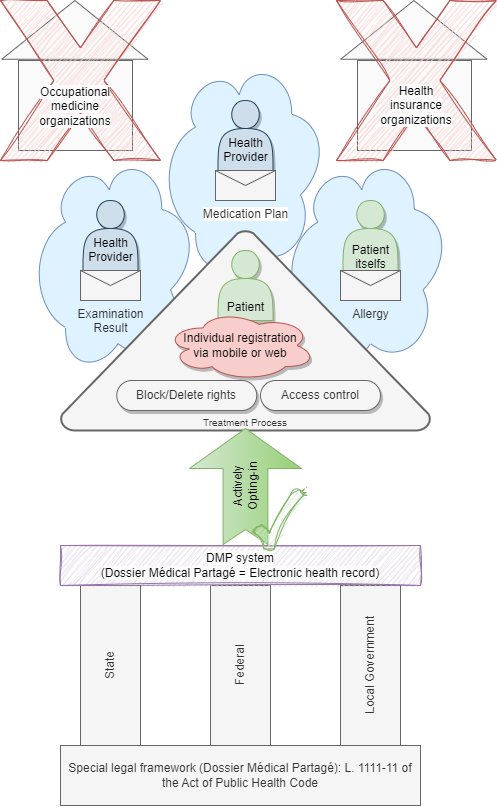


Figure S2: Systematic overview of EHR governance and exemplary access rights in France
