# Supplementary material for: How do opt-in versus opt-out settings nudge patients toward electronic health record adoption? An exploratory study of facilitators and barriers in Austria and France
Source: BMC Health Serv Res. 2024 Apr 8;24:439. doi: 10.1186/s12913-024-10929-w (PMC11003073; doi:10.1186/s12913-024-10929-w)
Supplement: Supplementary file 2 — Supplementary Material 2. [file 12913_2024_10929_MOESM2_ESM.docx]

Table S1: Interview Guideline

| **No usage experience**  **with electronic health records (EHR)** | **Usage experience with electronic health records (EHR)** |
| --- | --- |
| *Current Awareness REGARDING EHR* | |
| 1. What do you understand by electronic health records (EHR)? In Austria/France, EHR have been introduced as "electronic health records" | How long have you been using the EHR system? |
| 1. Are you familiar with the EHR system and have you had any contact with it in the past? (If yes: Can you tell us how you heard about it? and from whom? Have you ever tried to access the system? If yes: What was your experience? If no: Why not? Did you opt out? Why?) 2. What is your personal attitude towards the EHR system? | Can you tell us how you heard about it? and from whom? |
| 1. What do those around you think about the EHR system? Do you agree? Have you already had conversations about the system with a healthcare professional (e.g., doctor) or others (e.g., family/friends)? If so, what attitudes and different positions are you aware of here? Can you agree with these positions? If yes, why? If no, why not? What is your personal opinion? | Did you encounter any difficulties in accessing the EHR system? What could be improved?  What motivated you to access the EHR system?  How/in what way do you think the EHR system is useful? Why?  What do those around you think about the EHR system? Do you agree? |
| *Willingness to use EHR* | |
| 1. Do you think the information situation regarding the EHR system is sufficient? If not, why not? What would you like to see? In your opinion, is there a need for more information or training? | |
| Visualization of the system, with log-in, functions, etc. | |
| ***The visualization serves to ensure a comparable basic knowledge as an entry requirement of previous users and previous non-users, then identical procedure following the guideline*** | |
| *Usage experience with EHR* | |
| 1. After this presentation, do you feel sufficiently informed? If no, why not? What else would you like to see? Do you think that you trust the tool? Could you explain to us why? 2. How do you rate the user-friendliness of the EHR system? How practical do you think the EHR system is? 3. How/In what way do you think the EHR system is useful? Why? What are the benefits associated with the EHR system? Why? What do you think are the limits of the EHR system? Why? 4. What is your attitude towards the necessity of smartphone (FaceID, fingerprint), or digital forms of authentication? 5. [Non-users] What reasons do you have for not using (know-how)/   [and/or non-user] rejection (e.g.: data security) of the EHR system? Why have you not yet used the EHR system? In your opinion, can there be drifts to the EHR system and data sharing? How? or What? | |
| HEALTHCARE PROVIDERS | |
| 1. What do you think of medical confidentiality? Do you think that medical confidentiality still exists with the EHR system? 2. Do you know with whom your EHR is shared? What do you think about it? 3. Are you comfortable with the fact that your doctor ( + pharmacist, and other healthcare professionals, etc.) is able to know everything about your medical data? Would you share your data in the same way with all healthcare providers? Why? What would you like to share with each? Why? 4. Would you have more concerns about some healthcare providers than others? Which ones? Why? 5. Are you aware of any information material about the EHR system? If yes, which? How do you rate the existing information material on EHR? 6. Would you like to be informed more about the EHR system? How would you like to be informed about the EHR system? What would you like to see as information channels? (e.g.: social media stories, blog posts on websites, short videos on YouTube etc.). 7. How fit or competent do you feel in general with regard to dealing with the EHR system? | |
| COVID impact | |
| 1. Has your attitude toward EHR changed since the beginning of the COVID-19 pandemic? If so, in what form? | |
| *Conclusion* | |
| 1. Are there any other points/comments that you would like to make and that are on your mind? i.e. are there any points that you can think of with regard to the EHR system that you would otherwise like to introduce? | |
